# Supplementary material for: Learning plasma dynamics and robust rampdown trajectories with predict-first experiments at TCV
Source: Nat Commun. 2025 Oct 6;16:8877. doi: 10.1038/s41467-025-63917-x (PMC12501066; doi:10.1038/s41467-025-63917-x)
Supplement: Supplementary file 1 — Supplementary Information [file 41467_2025_63917_MOESM1_ESM.pdf]

Supplementary Information

Random Variable	Distribution (140kA/170kA scenarios)	Quantification Method
	**Disturbances**	
H-L back-transition threshold mult. factor	$N(\mu=1.15,\sigma=0.15)$ / $N(\mu=1.15,\sigma=0.1)$	Ad-hoc
Ohmic heating mult. factor	$N(\mu=1,\sigma=0.1)$ / $N(\mu=1,\sigma=0.1)$	Model prediction error
Radiated power mult. factor	$N(\mu=1,\sigma=0.5)$ / $N(\mu=1,\sigma=0.5)$	Model prediction error
Energy confinement time mult. factor	$N(\mu=1,\sigma=0.075)$ / $N(\mu=1,\sigma=0.075)$	Model prediction error
Particle confinement time mult. factor	$N(\mu=1,\sigma=0.075)$ / $N(\mu=1,\sigma=0.075)$	Model prediction error
Wall effects NN mult. factor	$N(\mu=1,\sigma=0.2)$ / $N(\mu=1,\sigma=0.2)$	Ad-hoc
γ_{vgr} mult. factor	$N(\mu=1,\sigma=0.2)$ / $N(\mu=1,\sigma=0.2)$	Model prediction error
g_{HFS} actuation mult. factor	$N(\mu=1,\sigma=0.5)$ / $N(\mu=1,\sigma=0.5)$	Database Control Errors
Limited τ_E mult. factor	$U(1,1.15)$ / $U(1,1.2)$	Ad-hoc
Limited τ_n mult. factor	$U(1,1.15)$ / $U(1,1.2)$	Ad-hoc
H-mode correction factor for τ_n	$U(0.3,0.5)$ / $U(0.3,0.5)$	Ad-hoc
Jump in κ at the diverted to limited transition	$U(-0.2,0.0)$ / $U(-0.2,0.0)$	Database Control Errors
	**L-mode Initial State**	
I_p	$N(\mu=1.4,\sigma=0.025)$ / $N(\mu=1.7,\sigma=0.04)$	Database Control Errors
W_{tot}	$N(\mu=10,0.5)$ / $N(\mu=12,0.5)$	Database Control Errors
$\bar{n}_{e,20}V$	$N(\mu=0.8,\sigma=0.05)$ / $N(\mu=0.8,\sigma=0.05)$	Database Control Errors
κ	$N(\mu=1.575,0.03)$ / $N(\mu=1.575,0.03)$	Database Control Errors
a_{minor}	$N(\mu=0.21,0.005)$ / $N(\mu=0.21,0.005)$	Database Control Errors
δ	$N(\mu=0.275,0.01)$ / $N(\mu=0.275,0.01)$	Database Control Errors
P_{NBI}	$U(1.05,1.15)$ / $U(1.1,1.2)$	Database Control Errors
	**H-mode Initial State**	
I_p	$N(\mu=1.4,\sigma=0.025)$ / $N(\mu=1.7,\sigma=0.04)$	Database Control Errors
W_{tot}	$N(\mu=12.5,0.5)$ / $N(\mu=16,0.5)$	Database Control Errors
$\bar{n}_{e,20}V$	$N(\mu=0.9,\sigma=0.05)$ / $N(\mu=1.1,\sigma=0.05)$	Database Control Errors
κ	$N(\mu=1.575,0.03)$ / $N(\mu=1.575,0.03)$	Database Control Errors
a_{minor}	$N(\mu=0.21,0.005)$ / $N(\mu=0.21,0.005)$	Database Control Errors
δ	$N(\mu=0.275,0.01)$ / $N(\mu=0.275,0.01)$	Database Control Errors
P_{NBI}	$U(1.05,1.15)$ / $U(1.1,1.2)$	Database Control Errors

Table S1. RL uncertainty model. The uncertainty model used in RL training environments to design trajectories with robustness to distributional uncertainty for both the 140kA and 170kA scenarios.

Category	Parameter	Value
Optimizer	Initial Learning Rate	0.02
	Final Learning Rate	0.0001
	Transition Steps	200
	Decay Rate	0.9
	Weight Decay	0.005
$NN_{ohm,rad}$	Depth	2
	Width	32
NN_{vgr}	Depth	3
	Width	256
NN_{conf}	Depth	1
	Width	128
NN_{prof}	Depth	2
	Width	64

Category	Parameter	Value
Hard Limits	f_{GW}	1.0
	ι_{95}	0.5
Soft Limits	f_{GW}	0.8
	β_p	1.75
	γ_{vgr}	0.75
	ι_{95}	0.313
Parameters	c_{time}	5.0
	c_{I_p}	1.0
	c_W	1.0
	c_{soft}	$1.0{\times}10^3$
	c_{hard}	$5.0{\times}10^4$

Table S2. Hyperparameters and reward function parameters. (Left) Hyperparameters for the final trained model. (Right) Reward function parameters. Note that the values corresponding to limits are the maximum allowed values.

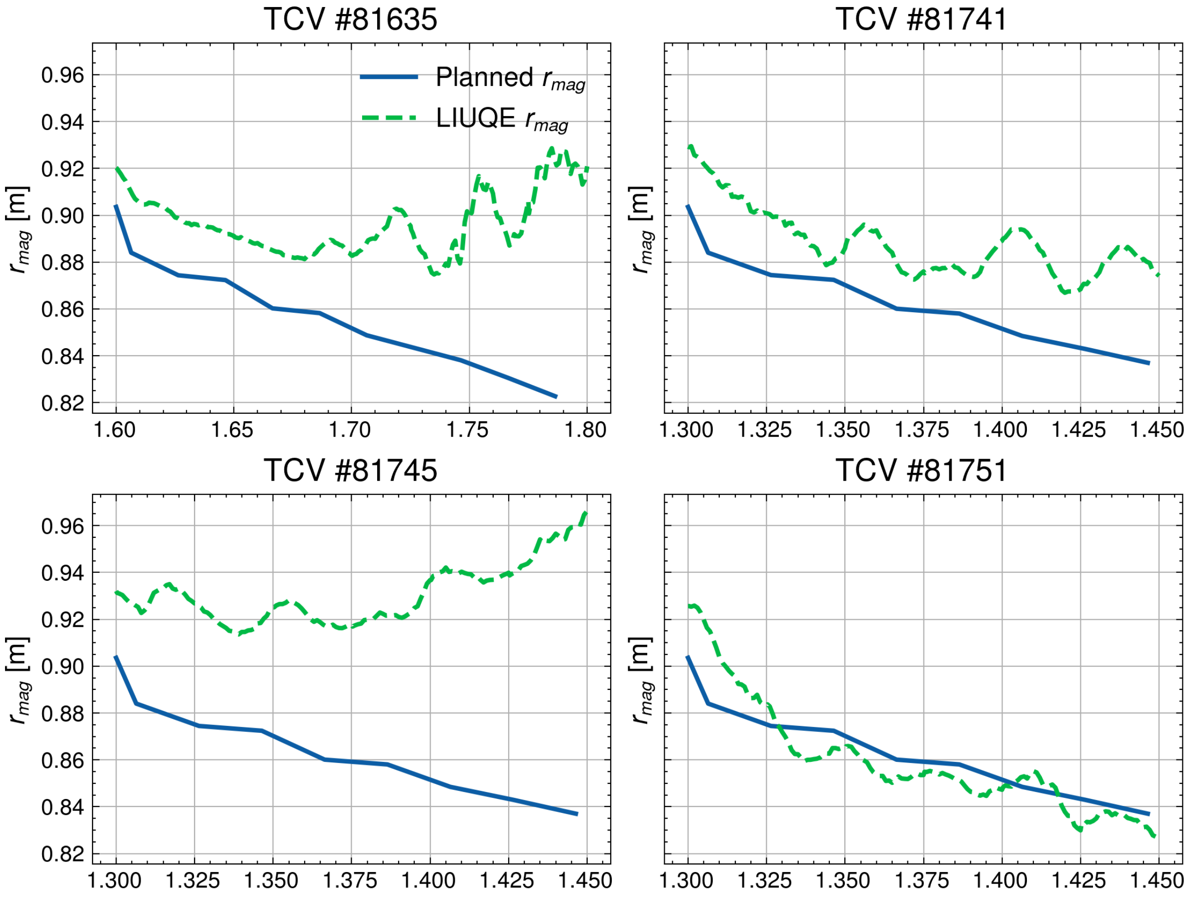

Figure S1. Debugging the legacy radial observer issue. Plot of planned radial magnetic position r_{mag} against observed values from equilibrium reconstruction with LIUQE, showing exceptionally poor control performance during initial rampdown attempts. The issue was identified as a legacy $\frac{1}{I_p^2}$ term used in generating the radial position observer feed-forward trajectory, with the issue finally fixed in TCV#81751.

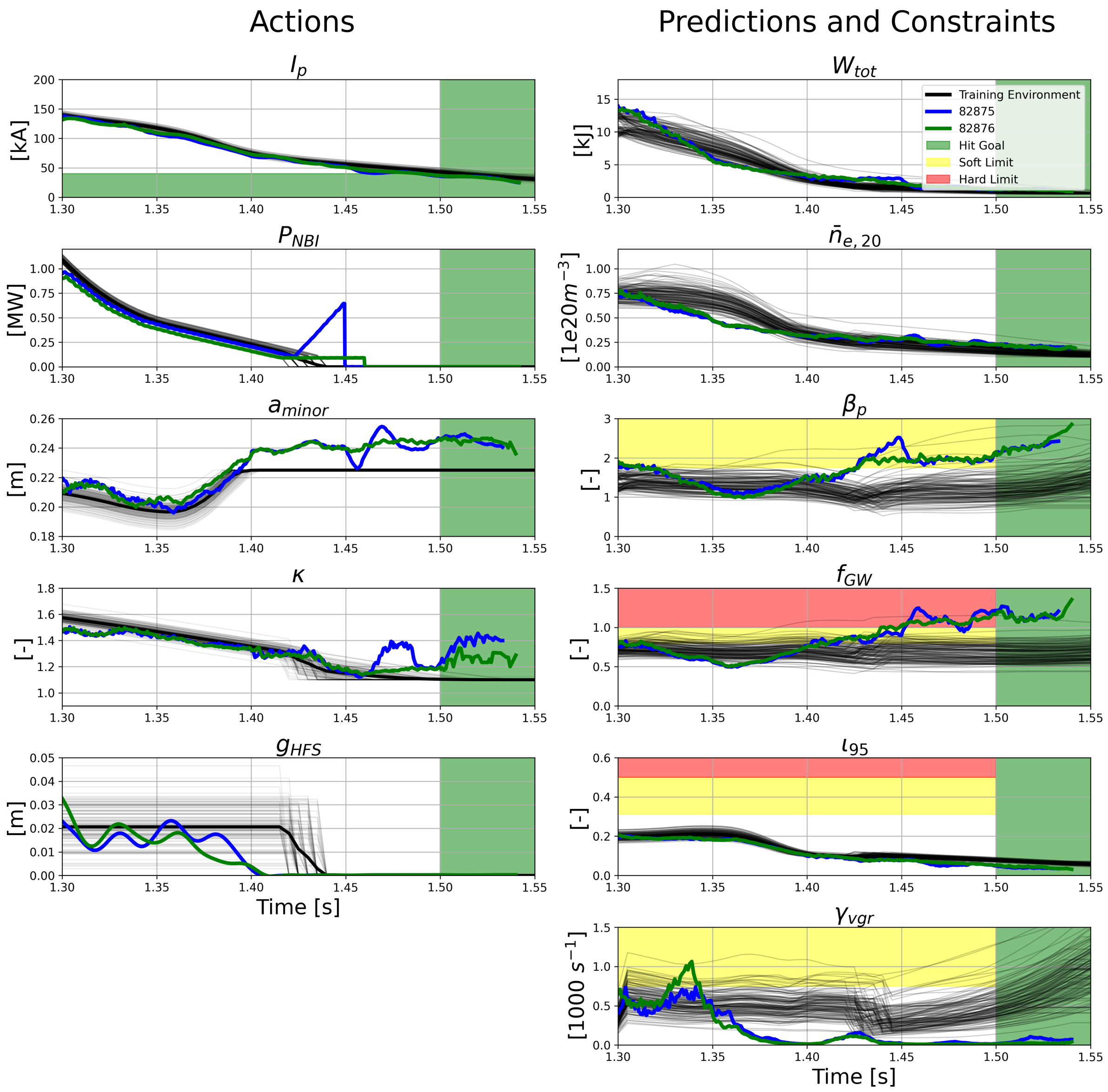

Figure S2. Results from the 140kA Scenario. Shown are a priori predictions of the RL environment and experimental outcomes of the final two 140kA shots.

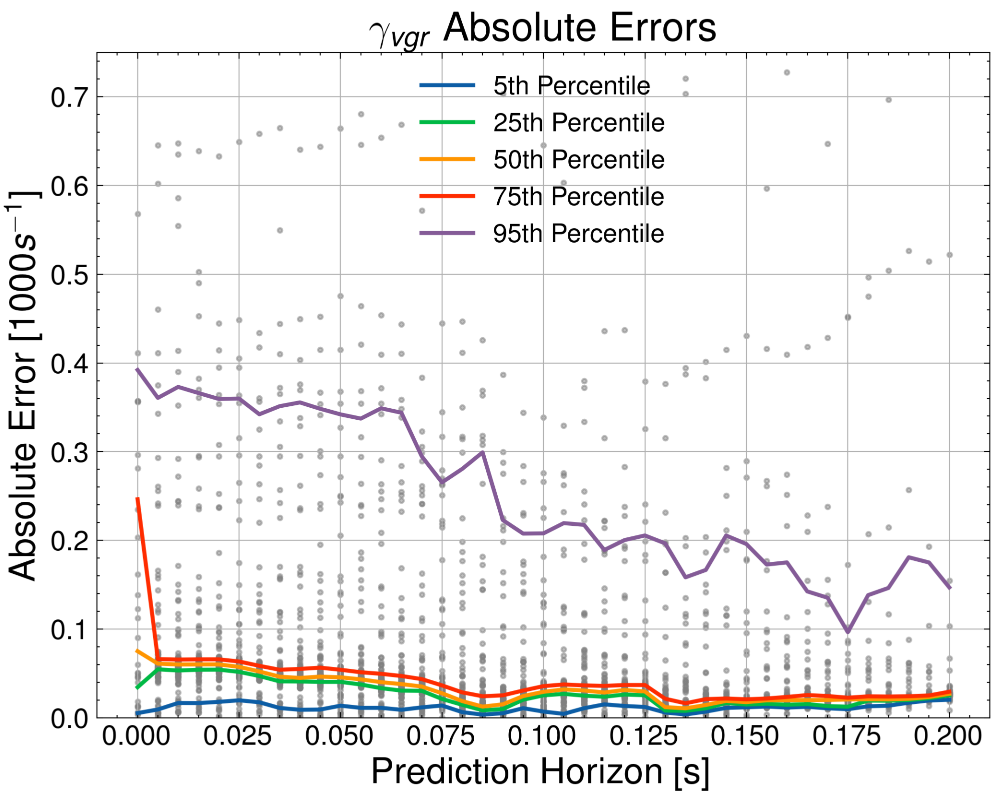

Figure S3. **Absolute errors for γ_{vgr}.** Errors are shown for the initial validation set of 131 shots, showing relatively low absolute prediction error in the majority of shots.

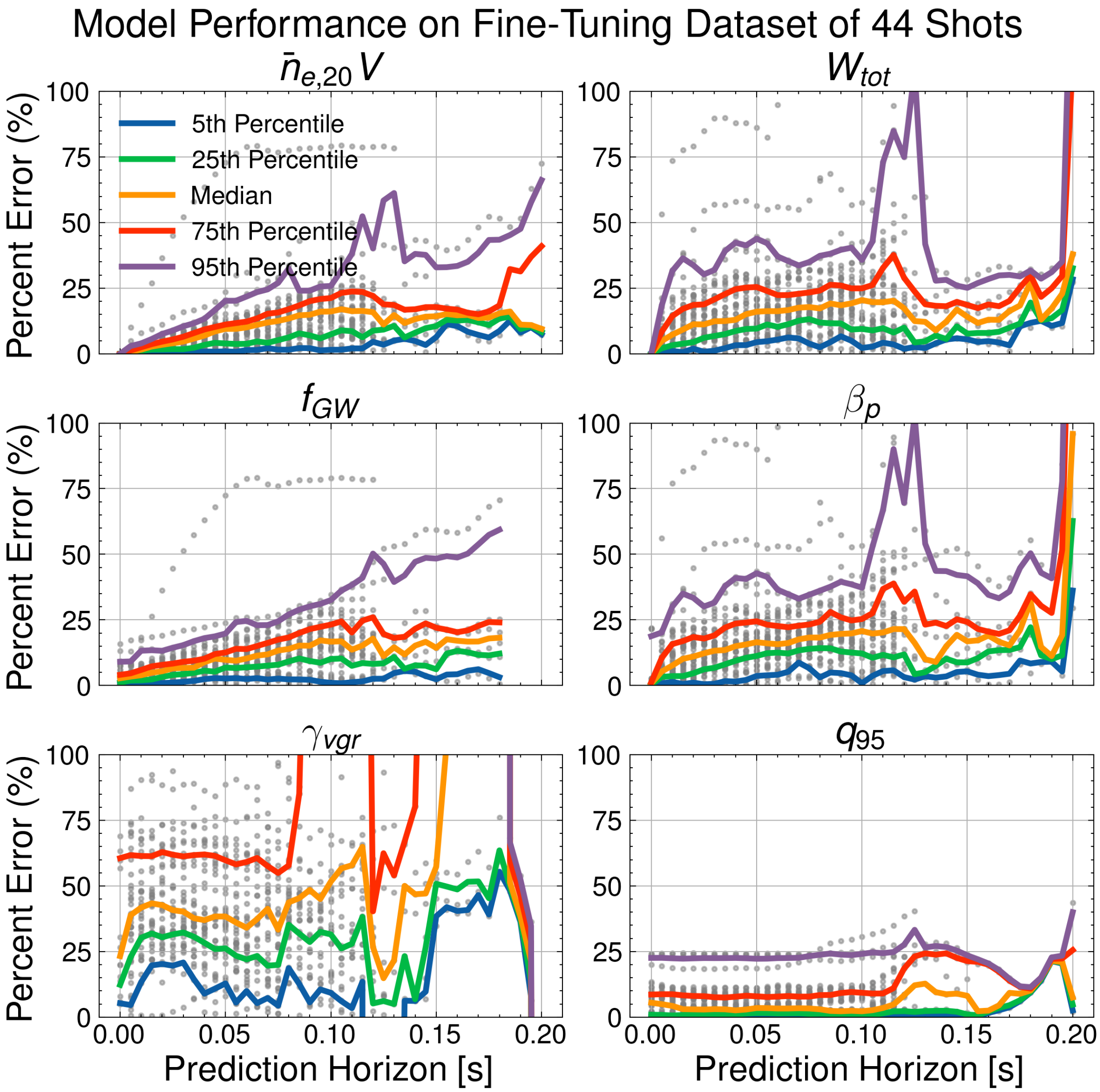

Figure S4. Fine-tuning dataset metrics. Model prediction accuracy as a function of prediction horizon during rampdowns on the fine-tuning dataset of 44 shots.

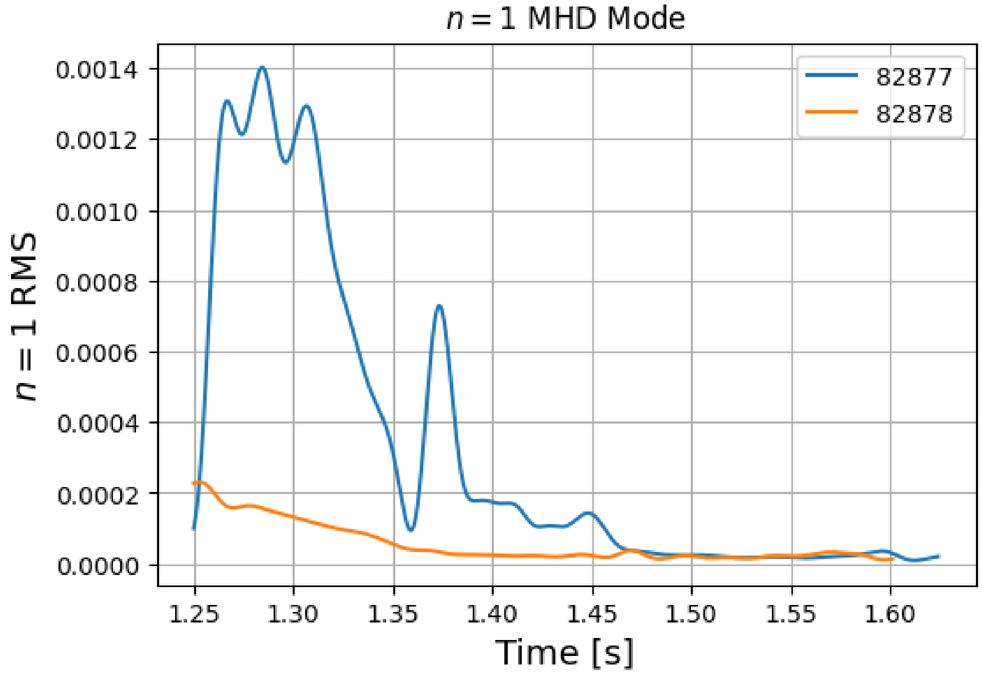

Figure S5. NTM driven discrepancy between #82877 and #82878. Shown is the $n = 1$ root mean squared (RMS) signal derived from magnetic diagnostics indicating the presence of a neo-classical tearing mode at the beginning of ramp-down for #82877 that is not present in #82878.

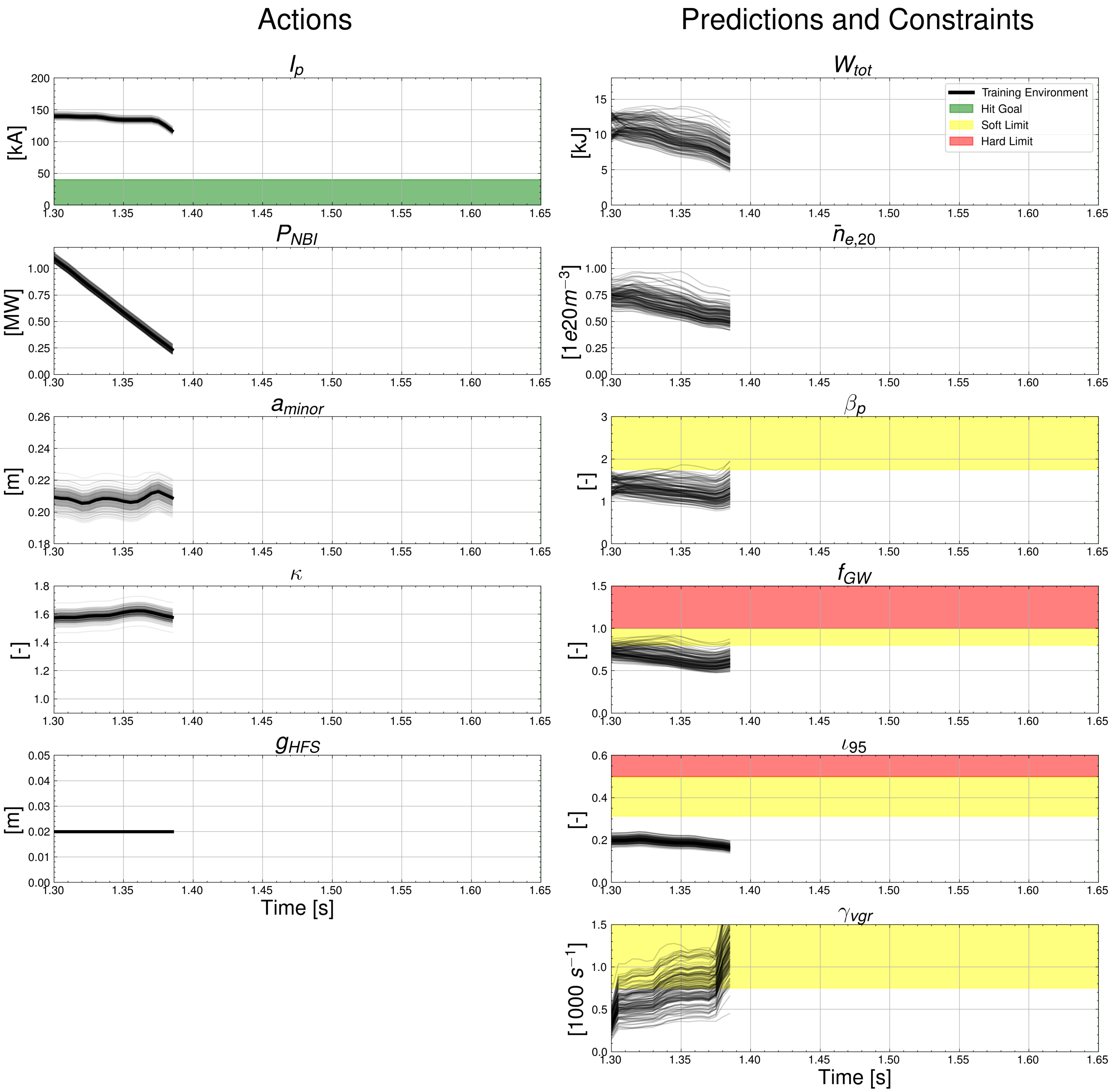

Figure S6. #81101 counterfactual analysis. A counterfactual analysis, using the action trajectories from the disruptive rampdown, #81101, in the RL training environment used for trajectory design. We see that the training environment predicts a large γ_{vgr}, well within the soft limit region right before the shot disrupted in experiment.

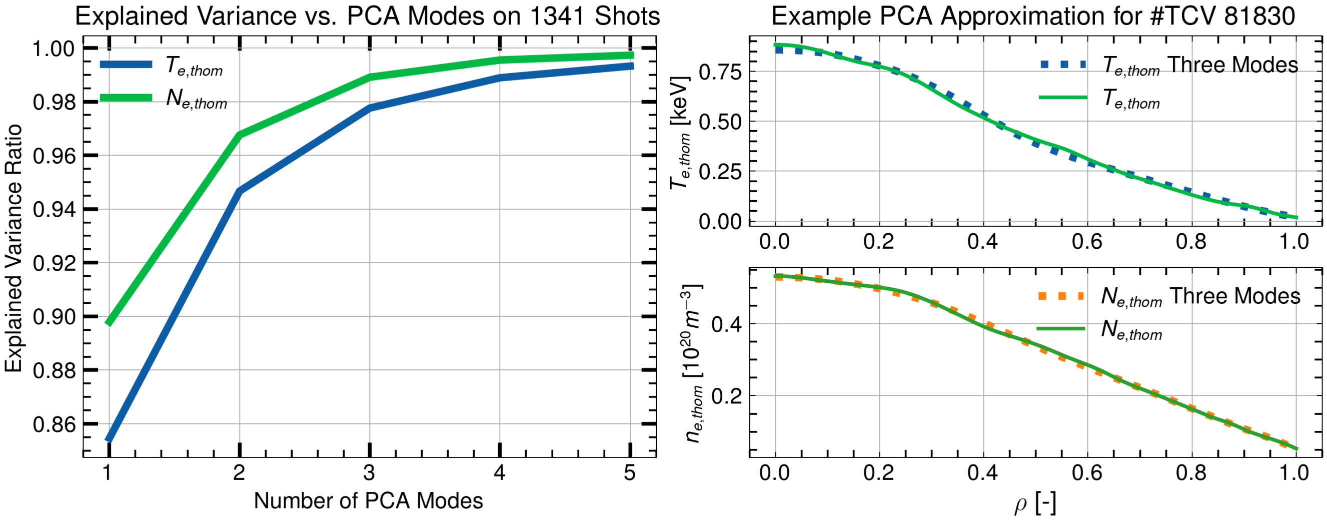

Figure S7. Principal component analysis (PCA) showing the empirical low dimensionality of kinetic profiles measured by Thomson Scattering. (Left) explained variance as a function of number of PCA modes, showing high explained variance with a relatively low dimensional representation of kinetic profiles on a database of 1341 shots. (Right) an example of kinetic profiles and their corresponding approximation with three PCA modes.

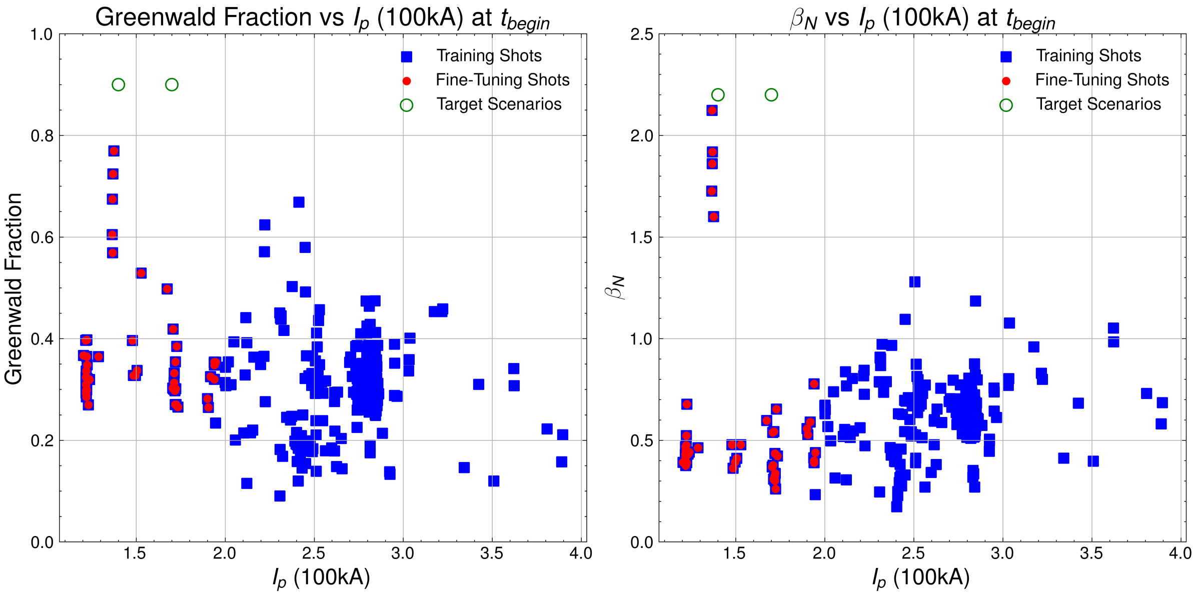

Figure S8. The data distribution used for training the NSSM. Each point corresponds to a shot, visualized in (I_p, f_{GW}) space and (I_p, β_N) space.

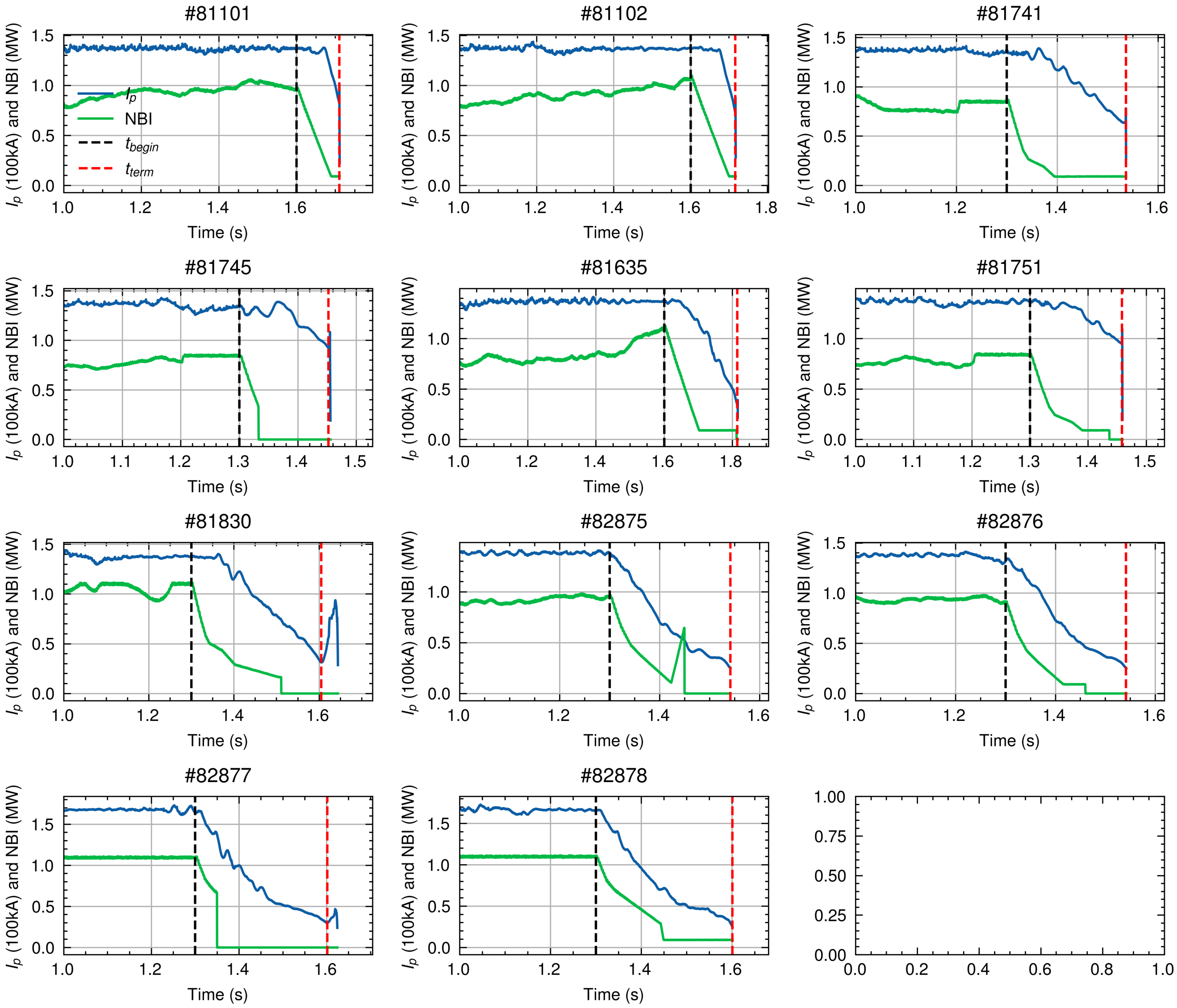

Figure S9. Definition of the beginning and end of the termination phase for every shot in this experiment. Note that in 81830, a low level software issue triggered an unexpected spike in plasma current at the very end of the rampdown. We define the termination time for that shot as the lowest achieved plasma current, as it is expected that net energy tokamaks where disruptions are of concern will have protection systems that prevent such incidents.

The TCV Team

Modified from: Duval, B. P., et al. "Experimental research on the TCV tokamak." Nuclear Fusion 64.11 (2024): 112023.

B.P. Duval[3], A. Abdolmaleki[5], M. Agostini[6], C.J. Ajay[7], S. Alberti[3], E. Alessi[8], G. Anastasiou[9], Y. Andrèbe[3], G.M. Apruzzese[10], F. Auriemma[6], J. Ayllon-Guerola[11], F. Bagnato[3], A. Baillod[3], F. Bairaktaris[12], L. Balbinot[6], A. Balestri[3], M. Baquero-Ruiz[3], C. Barcellona[13], M. Bernert[14], W. Bin[8], P. Blanchard[3], J. Boedo[15], T. Bolzonella[6], F. Bombarda[10], L. Boncagni[10], M. Bonotto[6], T.O.S.J. Bosman[16,45], D. Brida[14], D. Brunetti[17], J. Buchli[5], J. Buerman, P. Buratti[19], A. Burckhart[14], D. Busil[20], J. Caloud[21], Y. Camenen[22], A. Cardinali[10], S. Carli[23], D. Carnevale[19], F. Carpanese[3,5], M. Carpita[3], C. Castaldo[10], F. Causa[8], J. Cavalier[21], M. Cavedon[24], J.A. Cazabonne[3], J. Cerovsky[21], B. Chapman[17], M. Chernyshova[25], P. Chmielewski[25], A. Chomiczewska[25], G. Ciraolo[26], S. Coda[3], C. Colandrea[3], C. Contré[3], R. Coosemans[3], L. Cordaro[6], S. Costea[27], T. Craciunescu[28], K. Crombe[18,50], A. Dal Molin[8], O. D'Arcangelo[10,29], D. de Las Casas[5], J. Decker[3], J. Degrave[5], H. de Oliveira[3], G.L. Derks[16,45], L.E. di Grazia[29,51], C. Donner[5], M. Dreval[30], M.G. Dunne[14], G. Durr-Legoupil-Nicoud[3], B. Esposito[10], T. Ewalds[5], M. Faitsch[14], M. Farník[21], A. Fasoli[3], F. Felici[3], J. Ferreira[31], O. Février[3], O. Ficker[21], A. Frank[3], E. Fransson[32], L. Frassinetti[33], L. Fritz[5], I. Furno[3], D. Galassi[3], K. Gałązka[25,26], J. Galdon-Quiroga[11], S. Galeani[19], C. Galperti[3], S. Garavaglia[8], M. Garcia-Munoz[11], P. Gaudio[19], M. Gelfusa[19], J. Genoud[3], R. Gerru Miguelanez[34], G. Ghillardi[10], M. Giacomin[3], L. Gil[31], A. Gillgren[32], C. Giroud[17], T. Golfinopoulos[1], T. Goodman[3], G. Gorini[5,18], S. Gorno[3], G. Grenfell[14], M. Griener[14], M. Gruca[25], T. Gyergyek[27], R. Hafner[5], M. Hamed[16], D. Hamm[3], W. Han[1], G. Harrer[35], J.R. Harrison[17], D. Hassabis[5], S. Henderson[17], P. Hennequin[36], J. Hidalgo-Salaverri[11], J-P. Hogge[3], M. Hoppe[3,33], J. Horacek[21], A. Huber[5], E. Huett[3], A. Iantchenko[3], P. Innocente[6], C. Ionita-Schrittwieser[37], I. Ivanova Stanik[25], M. Jablczynska[25], A. Jansen van Vuuren[11], A. Jardin[38], H. Järleblad[34], A.E. Järvinen[39], J. Kalis[14], R. Karimov[3], A.N. Karpushov[3], K. Kavukcuoglu[5], J. Kay[5], Y. Kazakov[18], J. Keeling[5], A. Kirjasuo[40], J.T.W. Koenders[16,45], P. Kohli[5], M. Komm[21], M. Kong[3,17], J. Kovacic[27,52], E. Kowalska-Strzeciwilk[25], O. Krutkin[3], O. Kudlacek[14], U. Kumar[3], R. Kwiatkowski[41], B. Labit[3], L. Laguardia[8], E. Laszynska[25], A. Lazaros[12], K. Lee[3], E. Lerche[18], B. Linehan[1], D. Liuzza[10], T. Lunt[14], E. Macusova[21], D. Mancini[3,42], P. Mantica[8], M. Maraschek[14], G. Marceca[3], S. Marchioni[3], A. Mariani[8], M. Marin[3], A. Marinoni[1], L. Martellucci[19], Y. Martin[3], P. Martin[6], L. Martinelli[3], F. Martinelli[19], J.R. Martin-Solis[43], S. Masillo[3], R. Masocco[19], V. Masson[3], A. Mathews[3], M. Mattei[29], D. Mazon[26], S. Mazzi[3,26], S.Y. Medvedev[44], C. Meineri[6], A. Mele[29], V. Menkovski[45], A. Merle[3], H. Meyer[17], K. Mikszuta-Michalik[25], I.G. Miron[28], P.A. Molina Cabrera[3], A. Moro[8], A. Murari[6,53], P. Muscente[6,54], D. Mykytchuk[3], F. Nabais[31], F. Napoli[10], R.D. Nem[34], M. Neunert[5], S.K. Nielsen[34], A. Nielsen[34], M. Nocente[24], S. Noury[5], S. Nowak[8], H. Nyström[33], N. Offeddu[3], S. Olasz[46], F. Oliva[19], D.S. Oliveira[3], F.P. Orsitto[29], N. Osborne[47], P. Oyola Dominguez[11], O. Pan[14], E. Panontin[24], A.D. Papadopoulos[12], P. Papagiannis[12], G. Papp[14], M. Passoni[20], F. Pastore[3], A. Pau[3], R.O. Pavlichenko[30], A.C. Pedersen[34], M. Pedrini[3], G. Pelka[25], E. Peluso[19], A. Perek[3,16], C. Perez Von Thun[25], F. Pesamosca[3], D. Pfau[5], V. Piergotti[10], L. Pigatto[6], C. Piron[10], L. Piron[6,54], A. Pironti[29], U. Plank[14], V. Plyusnin[31], Y.R.J. Poels[3,45], G.I. Pokol[46], J. Poley-Sanjuan[3], M. Poradzinski[25], L. Porte[3], C. Possieri[19], A. Poulsen[34], M.J. Pueschel[16,45], T. Pütterich[14], V. Quadri[26], M. Rabinski[41], R. Ragona[34], H. Raj[3], A. Redl[42], H. Reimerdes[3], C. Reux[26], D. Ricci[8], M. Riedmiller[5], S. Rienäcker[36], D. Rigamonti[8], N. Rispoli[8], J.F. Rivero-Rodriguez[17], C.F. Romero Madrid[11], J. Rueda Rueda[11], P.J. Ryan[17], M. Salewski[34], A. Salmi[40], M. Sassano[19], O. Sauter[3], N. Schoonheere[26], R.W. Schrittwieser[37], F. Sciortino[14], A. Selce[8], L. Senni[10], S. Sharapov[17], U.A. Sheikh[3], B. Sieglin[14], M. Silva[3], D. Silvagni[14], B. Simmendefeldt Schmidt[34], L. Simons[3], E.R. Solano[48], C. Sozzi[8], M. Spolaore[6], L. Spolladore[19], A. Stagni[6,54], P. Strand[32], G. Sun[3], W. Suttrop[14], J. Svoboda[21], B. Tal[14], T. Tala[40], P. Tamain[26], M. Tardocchi[8], A. Tema Biwole[3], A. Tenaglia[19], D. Terranova[6,53], D. Testa[3], C. Theiler[3], A. Thornton[17], A.S. Thrysoe[34], M. Tomes[21], E. Tonello[3,20], H. Torreblanca[3], B. Tracey[5], M. Tsimpoukelli[5], C. Tsironis[12], C.K. Tsui[3,15], M. Ugoletti[6], M. Vallar[3], M. van Berkel[16], S. van Mulders[3,55], M. van Rossem[3], C. Venturini[3], M. Veranda[6,53], T. Verdier[34], K. Verhaegh[17], L. Vermare[36], N. Vianello[6,53], E. Viezzer[11], F. Villone[29], B. Vincent[3], P. Vincenzi[6], I. Voitsekhovitch[17], L. Votta[20], N.M.T. Vu[3,55], Y. Wang[3], E. Wang[49], T. Wauters[18], M. Weiland[14], H. Weisen[3], N. Wendler[25], S. Wiesen[49], M. Wiesenberger[34], T. Wijkamp[16,45], C. Wüthrich[3], D. Yadykin[32], H. Yang[26], V. Yanovskiy[21], J. Zebrowski[41], P. Zestanakis[9], M. Zuin[6,53] and M. Zurita[3].

[5]Google DeepMind, London, Ireland

[6]Consorzio RFX, Padova, Italy

[7]York Plasma Institute, University of York, Heslington, York, United Kingdom

[8]Istituto per la Scienza e Tecnologia dei Plasmi ISTP-CNR, Milano, Italy

[9]Aristotle University of Thessaloniki, Thessaloniki, Greece

[10]Unità Tecnica Fusione, ENEA, Frascati, Italy

[11]Universidad de Sevilla, Sevilla, Spain

[12]Department of Physics, National and Kapodistrian University of Athens, Athens, Greece

[13]Università degli Studi di Catania, Catania, Italy

[14]Max Planck Institute for Plasma Physics, Garching, Germany

[15]Center for Energy Research (CER), University of California-San Diego (UCSD), La Jolla, CA, USA

[16]DIFFER-Dutch Institute for Fundamental Energy Research, Eindhoven, Netherlands

[17]CCFE, Culham Science Centre, Abingdon, Oxon, United Kingdom
[18]Laboratory for Plasma Physics, LPP-ERM/KMS, Brussels, Belgium
[19]University of Rome Tor Vergata, Rome, Italy
[20]Politecnico di Milano, Milan, Italy
[21]Institute of Plasma Physics of the CAS, Prague, Czech Republic
[22]Aix-Marseille Université, CNRS, Marseille, France
[23]Department of Mechanical Engineering, KU Leuven, Leuven, Belgium
[24]Università di Milano-Bicocca, Milano, Italy
[25]Institute of Plasma Physics and Laser Microfusion (IPPLM), Warsaw, Poland
[26]CEA, IRFM, Saint-Paul-lez-Durance Cedex, France
[27]Jožef Stefan Institute, Ljubljana, Slovenia
[28]National Institute for Laser, Plasma and Radiation Physics, Magurele, Romania
[29]Università degli Studi di Napoli 'Federico II', Consorzio CREATE, Napoli, Italy
[30]Institute of Plasma Physics of the NSC KIPT, Kharkov, Ukraine
[31]Instituto de Plasmas e Fusão Nuclear, Instituto Superior Técnico, Lisboa, Portugal
[32]Chalmers University of Technology, Gothenburg, Sweden
[33]KTH Royal Institute of Technology, Stockholm, Sweden
[34]Department of Physics, Technical University of Denmark, Kgs, Lyngby, Denmark
[35]Institute of Applied Physics, Fusion at ÖAW, T.U. Wien, Vienna, Austria
[36]Laboratoire des Physique des Plasmas (LPP), Ecole polytechnique, Palaiseau, France
[37]Institut für Ionenphysik und Angewandte Physik, Universität Innsbruck, Innsbruck, Austria
[38]Institute of Nuclear Physics Polish Academy of Sciences (IFJ PAN), Krakow, Poland
[39]Aalto University, Aalto, Finland
[40]VTT, Espoo, Finland
[41]National Centre for Nuclear Research (NCBJ), Otwock, Poland
[42]Department of Economics, Engineering, Society and Business Organization (DEIm), University of Tuscia, Viterbo, Italy
[43]Universidad Carlos III de Madrid, Madrid, Spain
[44]Tokamak Energy, Abingdon, United Kingdom
[45]Eindhoven University of Technology, Eindhoven, Netherlands
[46]Centre for Energy Research, Budapest, Hungary
[47]University of Liverpool, Liverpool, United Kingdom
[48]Laboratorio Nacional de Fusión, CIEMAT, Madrid, Spain
[49]Forschungszentrum Jülich GmbH, Institut für Energie- und Klimaforschung—Plasmaphysik, Jülich, Germany
[50]Universiteit Gent, Ghent, Belgium
[51]Università degli Studi della Campania 'L. Vanvitelli', Aversa, Italy
[52]University of Ljubljana, Ljubljana, Slovenia
[53]Istituto per la Scienza e Tecnologia dei Plasmi ISTP-CNR, Padova, Italy
[54]Università degli Studi di Padova, Padova, Italy
[55]ITER Organization, Saint-Paul-lez-Durance, France
